# Specific alterations in gut microbiota in patients with chronic kidney disease: an updated systematic review

**DOI:** 10.1080/0886022X.2020.1864404

**Published:** 2021-01-06

**Authors:** Jin Zhao, Xiaoxuan Ning, Baojian Liu, Ruijuan Dong, Ming Bai, Shiren Sun

**Affiliations:** a Department of Nephrology, Xijing Hospital, The Fourth Military Medical University, Xi’an, China; b Department of Geriatrics, Xijing Hospital, The Fourth Military Medical University, Xi’an, China

**Keywords:** Chronic kidney disease, end-stage renal disease, gut microbiota, systematic review

## Abstract

**Background:**

Emerging evidence demonstrates that gut dysbiosis is implicated in the pathogenesis of chronic kidney disease (CKD) with underlying mechanisms involving mucosal and/or systematic immunity or metabolic disorders. However, the profile of gut microbiota in patients with CKD has not been completely explored.

**Methods:**

Databases from their date of inception to 31 March 2020 were systematically searched for case-control or cross-sectional studies comparing the gut microbial profiles in adult patients with CKD or end-stage renal disease (ESRD) with those in healthy controls. Quantitative analysis of alterations in gut microbial profiles was conducted.

**Results:**

Twenty-five studies with a total of 1436 CKD patients and 918 healthy controls were included. The present study supports the increased abundance of, phylum *Proteobacteria* and *Fusobacteria*, genus *Escherichia_Shigella*, *Desulfovibrio*, and *Streptococcus*, while lower abundance of genus *Roseburia*, *Faecalibacterium*, *Pyramidobacter*, *Prevotellaceae_UCG-001*, and *Prevotella_9* in patients with CKD; and increased abundance of phylum *Proteobacteria*, and genus *Streptococcus* and *Fusobacterium*, while lower abundance of *Prevotella*, *Coprococcus*, *Megamonas*, and *Faecalibacterium* in patients with ESRD. Moreover, higher concentrations of trimethylamine-N-oxide and p-cresyl sulfate and lower concentrations of short-chain fatty acids were observed. Gut permeability in patients with CKD was not determined due to the heterogeneity of selected parameters.

**Conclusions:**

Specific alterations of gut microbial parameters in patients with CKD were identified. However, a full picture of the gut microbiota could not be drawn from the data due to the differences in methodology, and qualitative and incomplete reporting of different studies.

## Introduction

Chronic kidney disease (CKD) is one of the most significant non-communicable diseases and carries a high social and economic burden [1]. The prevalence of CKD is relatively high, with values of, 10.8% in China [2], 13% in the United States (US) [3], and 10.2% in Norway [4]. Emerging evidence demonstrates that gut dysbiosis may be implicated in the pathogenesis of CKD with underlying mechanisms that may involve mucosal and/or systematic immunity and metabolic or neuroendocrine disorders [5,6]. The interaction between the intestine and kidney is referred to as the ‘gut-kidney axis’, in which the gut microbiota is an indispensable component [7].

**Figure 1.**
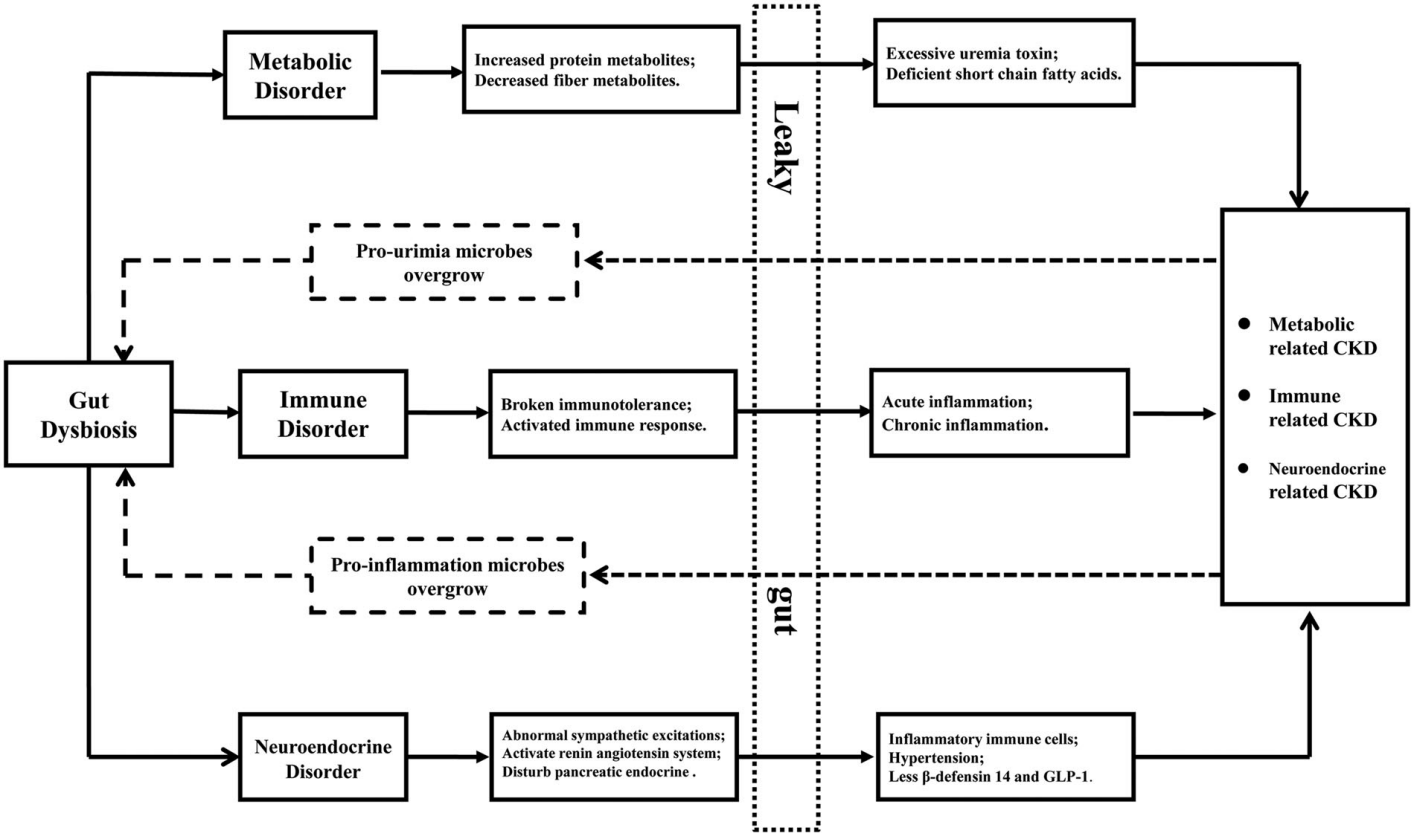
Vicious circle of gut dysbiosis and chronic kidney diseases. The solid line shows the effect of gut dysbiosis on kidney disease, while the dotted line shows the effect of kidney disease on intestinal flora. Metabolic related kidney disease mainly includes chronic kidney disease, diabetes nephropathy, and nephrolithiasis, etc. Immune related chronic kidney disease mainly includes IgA nephropathy, lupus nephritis, and diabetes nephropathy, etc. Neuroendocrine related kidney disease mainly includes diabetes nephropathy.

Gut dysbiosis is the condition of abnormal richness, evenness and composition of microbiota, which may contribute to immune, metabolic or endocrine disorders, causing or aggravating CKD [5]. Gut dysbiosis may result in imbalances between immune responses and immune tolerance, causing abnormal proliferation and differentiation of B and T lymphocytes with the production of autoantibodies and inflammatory factors that could contribute to CKD onset and progression [8,9]. Metabolites derived from gut microbiota, including the fermentation products of proteins or choline, such as trimethylamine-N-oxide (TMAO), p-cresyl sulfate (PCS), indoxyl sulfate (IS), and phenylacetylglutamine (PAG), may contribute to declining kidney function and worsening cardiovascular diseases, whereas short-chain fatty acids (SCFAs), fermentation products of dietary fiber, may exert protective effects on the kidney [10]. Excess uremic toxins are thought to promote the propagation of uremia-producing bacteria and to inhibit beneficial bacteria that produce SCFAs [11]. Moreover, gut microbiota have been implicated in neuroendocrine disorders that may also affect CKD. Gut dysbiosis can activate the local renin–angiotensin system in the kidney, triggering the initiation of diabetic nephropathy (DN) [12]. Conversely, SCFAs derived from the gut microbiota may stimulate glucagon-like peptide-1 secretion, which protects against chronic hyperglycemia induced by renal oxidative stress [13]. Gut dysbiosis impacts tight junctions and reduces the energy supplying of the colonic epithelium, increasing the permeability of the epithelium (so-called ‘leaky gut’) in CKD patients [14]. A ‘leaky gut’ allows translocation of bacteria and their products, along with immunogenic dietary antigens across the epithelium, activating local, and/or systemic inflammation [15]. The vicious circle of gut dysbiosis and CKD is shown in Figure 1. However, the profile of gut microbiota in patients with CKD has not been fully explored, and available studies are limited by relatively small sample sizes, geographical limitations, and methodological differences. Therefore, we performed a systematic review to analyze the bacterial diversity, relatively distinct bacterial taxa at different levels, bacterially derived metabolites, and gut permeability in patients with CKD compared with healthy individuals; these results may facilitate further research into the ‘gut-kidney axis’.

## Materials and methods

### Search strategy

We searched the literature in PUBMED, Web of Science, and The Cochrane Library on 31 March 2020 without language restriction. References of literature that had been selected were also screened for eligibility. The search strategy is provided in Table S1. Two researchers (JZ and MB) independently screened the articles by title, abstract and full text to determine eligibility. Inclusion criteria were case-control or cross-sectional studies describing gut microbial profiles of adult patients (>18 years of age) with CKD or end-stage renal disease (ESRD) and healthy controls. Exclusion criteria included studies confined to children or only kidney transplant recipients and studies with confounding factors or no control group.

### Quality evaluation

The Newcastle-Ottawa Scale [16] was applied to evaluate the quality of the selected case-control studies. The scale consisted of eight items that evaluated three dimensions, selection, comparability, and exposure, with scores between 0 and 10 indicating a gradual improvement in study quality.

### Data collection and statistics

Data were extracted into a pre-defined Excel sheet that included the following items: study characteristics, α-diversity, β-diversity, different bacterial taxa at various levels, bacterium-derived metabolites, and gut permeability. Data were extracted independently by JZ and MB, and discrepancies were solved by discussion and consensus. Uncertain or unpublished information was obtained by contacting the authors. Proportions (*n*/*N*) were used to present the alterations in gut microbiota profiles.

## Results

### Study selection, characteristics, and quality

**Figure 2.**
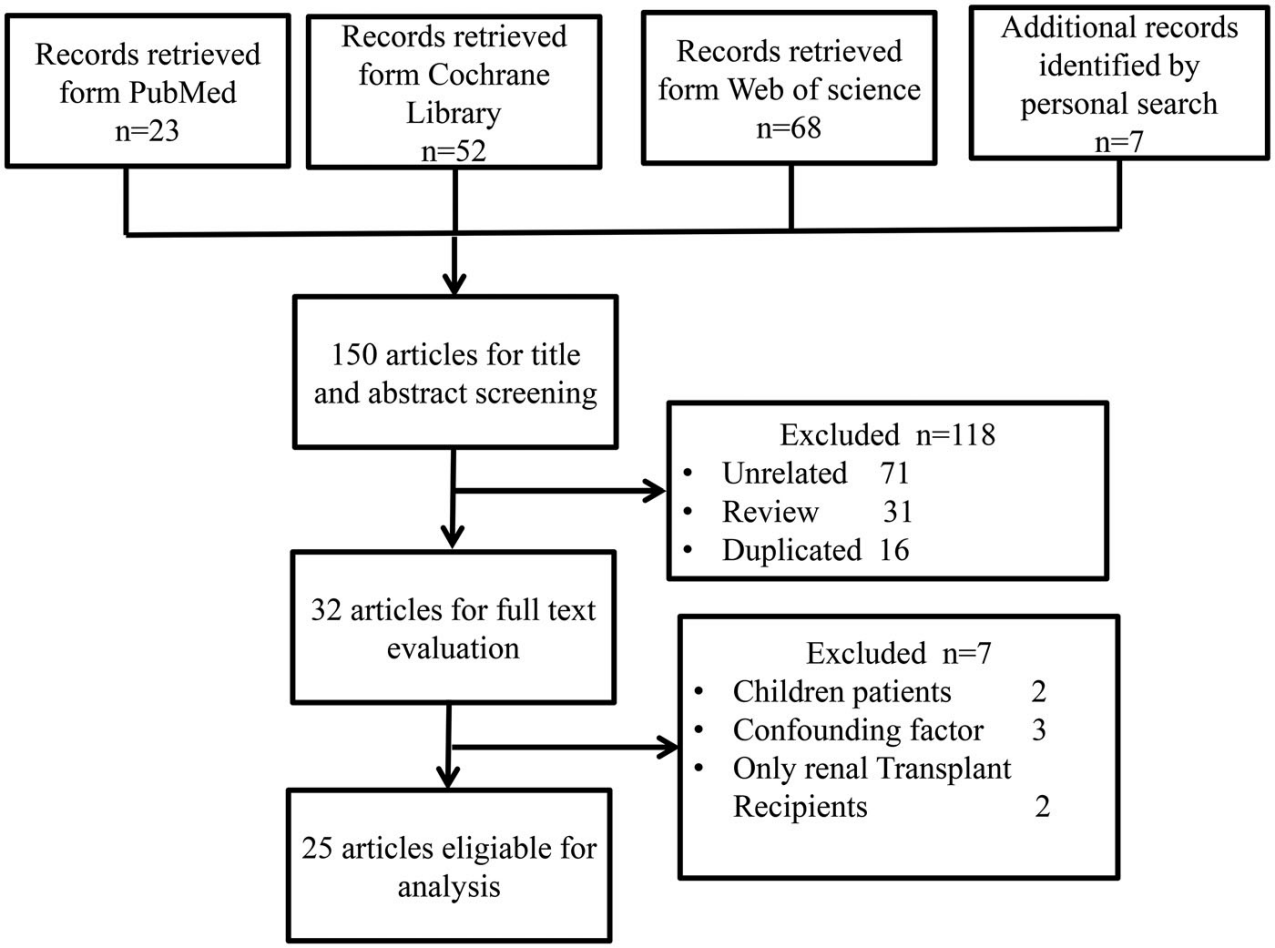
Study selection flowchart.

A total of 150 records were retrieved; 25, ranging from 2012 to 2020, were eligible for inclusion in our systematic review (Figure 2). The 25 studies included 1436 patients with CKD and 918 healthy controls (Table 1). Nine studies focused on ESRD; 12 focused on CKD ranging in severity from stage 1 to 5; and four focused on two specific pathological types of CKD: IgA nephropathy (IgAN) [17–19] and DN [20]. Seventeen studies were carried out in China, three in the US, two in Italy, and one in Austria, Brazil, and Netherlands. Nineteen studies conducted full-scale analysis of the gut microbial profile, while six were limited to specific bacteria at the species level and mainly applied polymerase chain reaction (PCR) analysis (Table S3). Patients in eight studies received renal replacement therapy including hemodialysis (*n* = 419), peritoneal dialysis (*n* = 68), or kidney transplantation (*n* = 20).

**Table 1.** Characteristics of studies included in this review.

Study	Year	Region	Center number	Disease/severity	*n* (case)	*n* (control)	Hemodialysis	Peritoneal dialysis	Kidney transplant	No dialysis
Wang et al.	2012	Shaanxi, China	1	ESRD	30	10	0	0	0	30
Wang et al.	2012	Taiwan, China	1	ESRD	29	41	0	29	0	0
Vaziri et al.	2013	California, USA	1	ESRD	24	12	24	0	0	0
Jiang et al.	2017	Guangdong, China	1	ESRD	52	60	0	0	0	52
Vanessa et al.	2017	Graz, Austria	1	ESRD	30	21	15	15	0	0
Ye et al.	2018	Guangdong, China	1	ESRD	100	53	84	0	16	0
Li et al.	2019	Shandong, China	1	ESRD	53	69	29	24	0	0
Terpstra et al.	2019	Amsterdam, Netherlands	1	ESRD	35	15	31	0	4	0
Wang et al.	2020	Beijing, China	4	ESRD	223	69	223	0	0	0
Total			12		575	350	406	68	20	82
Barros et al.	2015	Niterói, RJ, Brazil	1	CKD stage 3–4	20	19	0	0	0	20
Margiotta et al.	2020	Milano, Italy	1	CKD stage 3b–4	64	15	0	0	0	64
Xu et al.	2017	Guangdong, China	1	CKD stage 4–5	32	32	0	0	0	32
Salguero et al.	2019	Texas, USA	1	CKD stage 4–5 & T2DM	20	20	0	0	0	20
Al-Obaide et al.	2017	Texas, USA	1	CKD stage 4–5 & T2DM	20	20	0	0	0	20
Li et al.	2019	Shanxi, China	1	CKD stage 3–5	50	22	0	0	0	50
Jiang et al.	2016	Guangdong, China	1	CKD stage 1–5	65	20	0	0	0	65
Lun et al.	2018	Shandong, China	1	CKD stage 1–5	49	24	13	0	0	36
Wang et al.	2019	Guangdong, China	1	CKD stage 1–5	128	63	0	0	0	128
Wu et al.	2020	Taiwan, China	1	CKD stage 1–5	92	30	–	–	0	–
Hu et al.	2020	Hunan, China	1	CKD stage 1–5	95	20	0	0	0	95
Ren et al.	2020	Zhengzhou, China	1	CKD stage 1–5	110	210	0	0	0	110
Total			12		635	285	13	0	0	530
De Angelis et al.	2014	Bari, Italy	1	IgAN	32	16	0	0	0	32
Hu et al.	2020	Hunan, China	1	IgAN	17	18				17
Zhong et al.	2020	Sichuan, China	1	IgAN	52	25	0	0	0	52
Tao et al.	2019	Chengdu, China	1	DN	14	14	0	0	0	14
Total			28		1436	918	419	68	20	837

ESRD: end-stage renal disease; CKD: chronic kidney disease; T2DM: type 2 diabetes mellitus; IgAN: IgA nephropathy; DN: diabetic nephropathy.

All studies were conducted in adults.

Two studies were graded with seven stars, 22 studies were graded with six stars, and one study had five stars, suggesting a relatively high quality of the selected studies (Table S2).

### Sample collection and identification of gut taxa

Based on the concept that fecal microbiota may represent the gut microbiota, all studies collected and analyzed stool samples. 16S ribosomal RNA (16S rRNA) gene sequencing or metagenome shotgun sequencing was employed to analyze the full scale of the gut microbiota. PCR analysis was employed to identify specific microbes. Six (6/25) studies mentioned sample collection containers, whereas the other 19 studies did not (Table S3). Three (3/25) studies mentioned using special materials for preventing DNA degradation of microbes in fecal samples (Table S3).

### Alpha (α)-diversity

Thirteen (15/25) studies examined the α-diversity of fecal microbiota based on observed species, Chao1, phylogenetic diversity whole tree, Shannon, or Sobs indexes (Table S4), of which six focused on ESRD [21–27]. Six (6/9) and four studies (4/6) that included patients with CKD [19,23,25,27–29] and ESRD [23–26] demonstrated that the α-diversity of gut microbiota was significantly lower in patients than in healthy controls, respectively. Three (3/9) and two studies (2/6) that focused on CKD [17,30,31] or ESRD [21,22] suggested that α-diversity was similar in patients and healthy controls, respectively. Tao et al. [20] reported that the α-diversity in patients with DN was relatively higher than that in patients with type 2 diabetes mellitus without kidney disease (Table S4).

Of 15 studies, 11 studies were conducted in China, and four (4/11) studies focused on ESRD. Five (5/7) studies [18,27–29,32] carried in China showed that the α-diversity of gut microbiota was significantly lower in CKD patients than in healthy controls; one study [19] conducted in Italy showed that the α-diversity of gut microbiota in IgAN patients was significantly lower than in healthy controls, while another study [31] carried in Italy focused on CKD patients reported an opposite result. Three (3/4) studies [24–26] carried in China and one study [23] carried in Austria revealed that the α-diversity of gut microbiota was significantly decreased in ESRD patients than in healthy controls; while the study [21] conducted in the US reported a different result.

### Beta (β)-diversity

Seventeen (17/25) studies examined the β-diversity of fecal microbiota using parameters including principal coordinate analysis (PCA), principal component analysis (PC_O_A), non-metric multidimensional scaling (NMDS), and redundancy analysis, of which six studies focused on ESRD [21–26] (Table S5). Ten (10/11) studies that analyzed patients with CKD and five (5/6) studies that focused on ESRD documented that the composition of the gut microbiota in patients was statistically distant from that in healthy controls. One study in each subgroup suggested that the composition of gut microbiota in patients was similar to that in healthy controls [22,31].

Of 17 studies, 13 studies were conducted in China, and four (4/13) studies focused on ESRD. Nine (9/9) studies [17,18,20,27–30,32,33] carried in China showed that the β-diversity of gut microbiota in CKD patients was significantly distinct from that of healthy controls; one study [19] carried in Italy revealed that the β-diversity of gut microbiota in IgAN patients was significantly distinct from that of healthy controls, while another study [31] carried in Italy reported an opposite result. Three (3/4) studies [24–26] carried in China reported that the β-diversity of gut microbiota in the ESRD patients was significantly distinct from that of healthy controls. The studies carried in the US [21] and Austria [23] showed that the β-diversity of gut microbiota in the ESRD patients was significantly distinct from that in healthy controls.

### Relatively distinct bacterial taxa at the phylum level

Twelve (12/25) studies analyzed the relatively distinct taxa at the phylum level (Table S6), of which two studies (2/10) focused on ESRD [24,25]. Six (6/10) and three (3/10) studies demonstrated that *Proteobacteria* [24,25,27–29,33] and *Fusobacteria* [18,27,34] were more abundant, while two (2/10) studies [18,29] showed that the abundance of *Synergistetes* in patients with CKD was less than that in controls. The abundance of *Firmicutes*, *Bacteroidetes*, and *Actinobacteria* in patients with CKD was inconsistent when compared to controls. In the two studies [24,25] that focused on ESRD, *Proteobacteria* (2/2) was more abundant in patients than in controls, while the changes in the abundance of *Firmicutes* and *Bacteroidetes* were opposite in two studies. In total, seven (7/10) and three studies (3/10) showed *Proteobacteria* and *Fusobacteria* [18,25,34] were enriched in CKD patients, respectively.

Of 12 studies, 10 studies were conducted in China, and two (2/10) studies focused on ESRD. Five (5/8), two (2/8), and two (2/8) studies demonstrated that *Proteobacteria* [20,27–29,33], *Fusobacteria* [18,27], and *Bacteroidetes* [17,33] were more abundant, while four (4/8) and two (2/8) studies revealed that *Firmicutes* [17,27,28,33] and *Synergistetes* [18,29] were less abundant in Chinese CKD patients compared with healthy controls, respectively. However, the two studies conducted in the US [34] and Italy [19] did not identify any taxa with the same alteration in CKD patients. Two (2/2) studies carried in China showed that *Proteobacteria* [24,25] was more abundant in ESRD patients compared with healthy controls. However, studies conducted in the US [21], Austria [23], and Netherlands [35] did not report the distinct taxa at the phylum level in ESRD patients.

### Relatively distinct bacterial taxa at the class level

Eight (8/25) studies documented the relatively distinct taxa at the class level (Table S7), of which four (4/8) studies focused on ESRD, and seven (7/8) studies carried in China. In the ESRD subgroup, two (2/4) studies observed that *Deltaproteobacteria* [22,24], *Alphaproteobacteria* [23,25], and *Bacilli* [23,25] were more abundant in ESRD patients than in healthy controls. All the four studies focused on CKD were conducted in China, of which two (2/4) studies observed that *Actinobacteria* [27,30], *Gammaproteobacteria* [27,33], and *Fusobacteria* [17,27] were more abundant while *Verrucomicrobiae* [27,30] and *Betaproteobacteria* [27,30] were less abundant in CKD patients than in healthy controls, respectively.

### Relatively distinct bacterial taxa at the order level

Nine (9/25) studies reported relatively distinct taxa at the order level (Table S8). The nine studies were all conducted in China, of which three focused on ESRD. Two studies (2/3) reported an increased abundance of *Desulfovibrionales* [22,24] in ESRD patients compared with healthy controls. Three (3/6) and two (2/6) studies showed increased abundance of *Enterobacteriales* [27,28,33] and *Fusobacteriales* [17,27] in CKD patients compared with healthy controls, respectively; three (3/6), three (3/6), and two (2/6) studies reported decreased abundance of *Clostridiales* [17,27,28], *Burkholderiales* [27,28,30], and *Verrucomicrobiales* [27,30] in CKD patients compared with healthy controls. Additionally, *Desulfovibrionales* [27] was also enriched in a study focused on CKD.

### Relatively distinct bacterial taxa at the family level

Fourteen (14/25) studies documented the relatively distinct taxa at the family level (Table S9), of which five studies [21,23–25,28] focused on ESRD. Five (5/9) and two (2/9) studies showed that *Enterococcaceae* [18,19,27,28,33] and *Fusobacteriaceae* [17,27] were more abundant in patients with CKD than in healthy controls, respectively. Four (4/9) studies showed that *Prevotellaceae* [19,20,28,33] and *Lachnospiraceae* [27,28,32,33] were less abundant in patients with CKD than in healthy controls, respectively. Two (2/9) studies showed *Synergistaceae* [18,30] and *Lactobacillaceae* [19,30] were less abundant in patients with CKD than in healthy controls, respectively. Two (2/5) studies found that *Enterococcaceae* [21,25], *Streptococcaceae* [23,25], and *Desulfovibrionaceae* [22,24] were more abundant in patients with ESRD than in controls. Three (3/5), two (2/5), and two (2/5) studies found that *Prevotellaceae* [22,24,30], *Veillonellaceae* [24,25], and *Ruminococcaceae* [24,25] were less abundant in patients with ESRD than in controls, respectively.

Of the 14 studies, 10 (11/14) studies were conducted in China, and eight studies focused on CKD. Four (4/8) and two (2/8) studies revealed that *Enterococcaceae* [18,27,28,33] and *Fusobacteriaceae* [17,27] were more abundant in patients with CKD than in healthy controls, respectively. However, four (4/8) and three (3/8) studies reported that *Lachnospiraceae* [27,28,32,33] and *Prevotellaceae* [20,28,33] were less abundant in patients with CKD than in healthy controls, respectively. Two (2/3) studies found *Desulfovibrionaceae* [22,24] were more abundant in patients with ESRD than in controls. Three (3/3), two (2/3), and two (2/3) studies found that *Prevotellaceae* [22,24,30], *Veillonellaceae* [24,25], and *Ruminococcaceae* [24,25] were less abundant in patients with ESRD than in controls, respectively. However, studies conducted in the US [21], Austria [23], and Italy [19] did not identify the distinct taxa at the family level in CKD or ESRD patients.

### Relatively distinct bacterial taxa at the genus level

Sixteen (16/25) studies reported relatively distinct taxa at the genus level (Table S10), of which five (5/16) studies [21–25] focused on ESRD. Six (7/11), three (3/11), three (3/11), and two (2/11) studies showed that *Escherichia Shigella* [17,18,20,27,32,33,36], *Desulfovibrio* [27,30,36], *Bacteroides* [17,32,33], and *Streptococcus* [27,36] were more abundant, and five (5/11), three (3/11), two (2/11), and two (2/11) studies reported that *Roseburia* [27,28,31–33], *Pyramidobacter* [18,29,30], *Bifidobacterium* [17,36], and *Prevotellaceae_UCG-001* [18,29] were less abundant in patients with CKD than in healthy controls, respectively. Two (2/5) studies showed that *Streptococcus* [23,25] and *Fusobacterium* [22,25] were more abundant in ESRD patients than in controls. Three (3/5) studies observed that *Prevotella* [22–24], *Coprococcus* [22–24], *Megamonas* [22,24,25], and *Faecalibacterium* [22,24,25] were less abundant in patients with ESRD than in healthy controls.

Of the 16 studies, 13 (13/16) studies were conducted in China, and nine studies focused on CKD. Six (6/9), three (3/9), two (2/9), and two (2/9) studies showed that *Escherichia Shigella* [17,18,20,27,32,33], *Bacteroides* [17,32,33], *Desulfovibrio* [27,30], and *Lachnoclostridium* [17,32] were more abundant, while four (4/9), three (3/9), three (3/9), two (2/9), and two (2/9) studies reported that *Roseburia* [27,28,32,33], *Faecalibacterium* [22,25,27], *Pyramidobacter* [18,29,30], *Prevotellaceae_UCG-001* [18,29], and *Prevotella_9* [20,37], were less abundant in patients with CKD than in controls, respectively. Two (2/4) studies showed *Fusobacterium* [22,25] were more abundant in ESRD patients than in controls. Three (3/4), three (3/4), two (2/4), and two (2/4) studies observed that *Megamonas* [22,24,25], *Faecalibacterium* [22,24,25], *Prevotella* [22,24], and *Coprococcus* [22,24] were less abundant in patients with ESRD than in healthy controls, respectively. The studies conducted in Austria [23] and the US [21] focused on ESRD patients did not identify the same taxa with alteration in the same direction, neither did the two studies on CKD patients which were conducted in Italy [31] and the US [36].

### Relatively distinct bacterial taxa at the species level

Ten (10/25) studies identified microbes at the species level (Table S11), of which five (5/10) studies [15,26,35,38,39] focused on ESRD. *Prevotella* spp [26,39] (2/5), *Faecalibacterium* [26,39] (2/5), and *Bifidobacterium* [38,39] (2/5) were less abundant in patients with ESRD than in controls. *Escherichia coli* [19,32] (2/5) was more abundant in CKD patients than in controls. Additionally, *Faecalibacterium prausnitzii* [26,40] (2/10) and *Bifidobacterium* [19,38] (2/10) were less abundant, while *Escherichia spp* [15,19]. (2/10) was more abundant in patients within two subgroups compared with controls. However, the abundance of *Roseburia* spp. in CKD patients [19,31,40] and ESRD patients [26,35,39] was not consistent.

Of the 10 studies, six studies were conducted in China, and four studies (4/6) focused on ESRD patients. *Roseburia* spp [26,39] (2/4) and *Bifidobacterium* [38,39] (2/4) were less abundant in ESRD patients compared to healthy controls. The studies conducted in Netherlands [35] and Brazil [41] focused on ESRD patients did not identify the same taxa with alteration in the same direction, neither did the two studies on CKD patients which were conducted in Italy [19,31].

### Metabolites derived from gut microbiota

Five studies (5/25) analyzed the metabolites derived from gut microbiota between CKD patients and healthy controls (Table S12). Four (4/4) studies [26,28,36,39] found higher serum levels of TMAO, and one study [26] found higher feces levels of TMAO, in advanced CKD patients compared with healthy controls. Two (2/2) studies [26,32] found higher level of PCS in patients compared with healthy controls. Two (2/2) studies [26,39] reported lower level of SCFAs in advanced CKD patients.

### Gut permeability of patients with advanced CKD

Four studies (4/25) analyzed the gut permeability of patients with advanced CKD by measuring correlated markers, serum d-lactate and Zonulin (Table S13). Two studies (2/2) confirmed higher d-lactate concentrations in patients with ESRD [15,35]. However, the serum level of Zonulin was not consistent due to opposite results from two studies [23,36].

## Discussion

We summarized the existing evidence describing the profile of gut microbiota in patients with CKD or ESRD with the aim of identifying specific microbial taxa that could contribute to disease pathogenesis or progression and that could form the basis for new approaches to modulate gut dysbiosis to treat and prevent CKD. Of note, these studies were mainly carried out in China (17/25), and some parameters were shown only in studies conducted in China. Thus, we should prudently employ the results from our present study in patients who are not from China. However, the microbial taxa with same alterations, which may be specific to CKD, were identified through comparison of the available data from all selected studies. The concentrations of TMAO and PCS were increased, whereas the concentration of SCFAs was decreased in patients with CKD. Similar systematic reviews [42,43] were conducted previously; however, the specific alternations in gut microbiota profiles in patients with CKD were not fully explored, and more papers have since been published. To aid in the interpretation of the data, we defined a parameter as altered if it changed in the same direction in more than two studies and if there was no available study reporting an opposite result. The alterations in gut microbial profiles in patients with CKD or ESRD were determined and are shown in Table 2.

**Table 2.** Characteristics of intestinal microbiota of patients with CKD and ESRD compared to healthy controls.

Characteristics of intestinal microbiota of patients with CKD		
Alpha diversity	66.6% (6/9) studies showed lower richness compared to healthy controls	
Beta diversity	90.9% (10/11) studies showed distinct bacterial composition from healthy controls	
Alteration of taxa	More abundant	Less abundant
Phylum	Proteobacteria and Fusobacteria	Synergistetes
Class	Bacteroidia, Gammaproteobacteria, Fusobacteria, and Actinobacteria	Betaproteobacteria and Verrucomicrobiae
Order	Enterobacteriales and Coriobacteriales	Clostridiales, Burkholderiales, and Verrucomicrobiales
Family	Enterococcaceae and Fusobacteriaceae	Prevotellaceae, Lachnospiraceae, Synergistaceae, and Lactobacillaceae
Genus	Escherichia Shigella, Desulfovibrio, and Streptococcus	Roseburia, Faecalibacterium, Pyramidobacter, Prevotellaceae_UCG-001, and Prevotella_9
Species	*Escherichia coli*	
Characteristics of intestinal microbiota of patients with ESRD		
Alpha diversity	66.7% (4/6) studies showed lower richness compared to healthy controls	
Beta diversity	83.3% (5/6) studies showed distinct bacterial composition from healthy controls	
Alteration of taxa	More abundant	Less abundant
Phylum	Proteobacteria	–
Class	Alphaproteobacteria, Deltaproteobacteria, and Bacilli	Betaproteobacteria
Order	Desulfovibrionales	–
Family	Enterococcaceae, Streptococcaceae, and Desulfovibrionaceae	Prevotellaceae, Ruminococcaceae, and Veillonellaceae
Genus	Streptococcus and Fusobacterium	Prevotella, Coprococcus, Megamonas, and Faecalibacterium
Species		*Prevotella* spp., Faecalibacterium, and Bifidobacterium

ESRD: end-stage renal disease; CKD: chronic kidney disease.

Although two opposite outcomes were observed, the composition (beta-diversity) of gut microbiota in patients with CKD or ESRD may be obviously changed compared with healthy controls. Meanwhile, lower richness (alpha-diversity) was also exhibited in more than half of the studies; together, these results may present the condition of gut dysbiosis in CKD patients. Phylum *Proteobacteria* was enriched in patients with CKD and ESRD than in healthy controls, which is consistent with the profile of blood microbiota in patients with CKD [44], suggesting bacterial translocation from the intestine to blood. Sputum *Proteobacteria* was also associated with bronchiectasis severity [45]. Gamma-*Proteobacteria* was enriched in new-born mice and depleted in normal adult microbiota, which was regulated by a gamma-*Proteobacteria*-specific IgA response, thus higher abundance of gamma-*Proteobacteria* was associated with sustained intestinal inflammation [46] which may also perpetrate kidney injury. Phylum *Fusobacteria* and genus *Fusobacterium* were expanded in patients with CKD, and were also enriched in patients with colorectal cancer [47] and chronic hepatitis B disease [48]. The potential ability of *Fusobacterium* for inhibiting T-cell proliferation and inducing T-cell apoptosis may perpetrate local or system immune disorder [47] leading to immune-mediated kidney diseases. A Mendelian randomization analysis of data from genome-wide association studies (GWAS) showed that people with higher abundance of *Desulfovibrio* spp. had a significantly lower level of estimated glomerular filtration rate (eGFR) [49], which may support the higher abundance of *Desulfovibrio* in CKD patients. Acute post-streptococcal glomerulonephritis is a post-infectious immune-mediated kidney disease associated with group A *Streptococcus* [50], and enriched gut abundance of *Streptococcus* in CKD patients may also imply the association between kidney injury and *Streptococcus* mediated immunity disorder. Uropathogenic *Escherichia coli*, the leading pathogen of urinary tract infections (UTIs) or hemolytic uremic syndrome, contributes to kidney injury through alpha-hemolysin or Shiga toxin [51], and a 1% relative gut abundance of *Escherichia* is an independent risk factor for *Escherichia* bacteriuria and UTI [52]. Depletion of the genera *Prevotella*, *Roseburia*, *Faecalibacterium*, and *Coprococcus* may result in less production of butyrate [22] which is known to have renal protection effects [53]. Genus *Bifidobacterium* was depleted in CKD patients, and supplementing *Bifidobacterium longum* reduced serum creatinine, urea nitrogen, and PCS in CKD models [54]. However, probiotic supplementation (containing *Bifidobacterium longum*) did not generate positive effect in ESRD patients who underwent maintenance hemodialysis [55]. *Megamonas* was also depleted in patients with cardiac valve calcification [56] and heart failure [57], the critical complications of CKD, potentially implying the same underlying mechanism. The above evidence suggests that the altered bacterial taxa may specific to CKD and/or its complications. However, gut dysbiosis and CKD may interact as both cause and effect, and the mechanism by which these altered bacterial taxa or micro-ecological imbalance play a role in the progression of CKD warrants further investigation.

Higher levels of TMAO and PCS and lower levels of SCFAs were observed in patients with CKD or ESRD, which was consistent with a study indicating that bacteria possessing urease-, urase-, indole-, and para-cresol-forming enzymes expanded significantly while those possessing butyrate-forming enzymes were depleted in patients with ESRD [11]. TMAO, a product of choline or l-carnitine through fermentation by gut microbiota in the colon [58], was also positively correlated with adverse cardiovascular events [59], hypertension [60], and diabetes mellitus [61]. Endothelial dysfunction is thought to be the underlying mechanism by which TMAO may contribute to the progression of CKD [58]. In addition, blocking the production of gut microbiota derived TMAO may alleviate the kidney lesions [62]. PCS, the product of aromatic amino acids (like tyrosine and phenylalanine), was identified as an independent risk factor for renal progression and all-cause mortality in adult patients with CKD in a prospective observational study [63]. Moreover, modulation of gut microbiota would decrease the serum PCS and other uremic toxins in CKD [64], which, to some extent, verified the relationships between gut dysbiosis and CKD. SCFAs, mainly acetate, propionate, and butyrate, are the primary energy source of epithelial cells, which provide approximately 10% of the daily caloric requirement in humans [65]. To date, SCFAs have been identified as beneficial products derived from gut microbiota that exert protective effects in AKI [66], CKD [40], DN [67], and hypertension [68] via interplay with olfactory receptor (Olfr) 78, G-protein-coupled receptor (GPCR) 41 or GPCR43.

The alteration of gut permeability in patients with CKD remains unclear due to fewer studies and their controversial results concluded from different markers (d-lactate and Zonulin). Bacterial translocation was detected in some ESRD patients whose serum d-lactate levels were concurrently increased, suggesting that the gut permeability of ESRD patients was higher. d-Lactate, a fermentation product of gut microbes [69], does not easily pass the intestinal barrier and enter into the circulation system. Thus, the concentration of serum d-lactate is very low in healthy populations, and would increase in blood through translocation across the aberrant gut barrier in patients. However, an increase in serum d-lactate was not observed in pediatric patients with ESRD [70] and patients on hemodialysis [69]. Meanwhile, Zonulin may perturb the actin cytoskeleton and cell–cell junctions in the gut epithelium by activating epidermal growth factor receptor (EGFR) through protease-activated receptor 2 (PAR2), and GPCR2 [71]. Increased plasma Zonulin was associated with nephrotic syndrome in children regardless of the quantity of proteinuria or therapeutic regimen [72]. However, one of the included studies showed that Zonulin was not elevated in patients with CKD [23]. Lukaszyk et al. also observed that Zonulin was significantly lower in patients with early-stage CKD than in healthy individuals, and was not associated with the state of inflammation [73]. The hemodialysis patients whose serum d-lactate was not elevated found increased serum Zonulin [69]. Therefore, the physiological role of Zonulin and the real situation of gut permeability need further elucidation.

We did not conduct a meta-analysis because of the significant heterogeneity between the selected studies and the qualitative reporting of results. Moreover, gut flora cannot be identified at the species level by 16S rRNA sequencing, and the number of species that can be detected at one time by PCR is limited, leading to a lack of integration of the available data. Drugs and/or dietary restriction for different stages of CKD, as well as the underlying kidney disease, may also affect the intestinal microbiome, which could not be identified in the present study.

## Conclusions

The present study supports the increased abundance of, phylum *Proteobacteria* and *Fusobacteria*, genus *Escherichia_Shigella*, *Desulfovibrio*, and *Streptococcus*, while lower abundance of genus *Roseburia*, *Faecalibacterium*, *Pyramidobacter*, *Prevotellaceae_UCG-001*, and *Prevotella_9* in patients with CKD; and increased abundance of phylum *Proteobacteria*, and genus *Streptococcus* and *Fusobacterium*, while lower abundance of *Prevotella*, *Coprococcus*, *Megamonas*, and *Faecalibacterium* in patients with ESRD. Moreover, higher concentrations of TMAO and PCS but lower concentrations of SCFAs were observed in advanced CKD patients. However, the characteristics of the gut microbiota in patients with CKD were not determined due to the heterogeneity of the available data. Further investigations should employ high-throughput sequencing technology and conduct comprehensive reporting to facilitate an in-depth understanding of gut microbiota, which could help decipher the underlying mechanisms of how the gut microbiota interact with CKD and its potential implication in CKD for disease treatment and prevention.

## Supplementary Material

Supplemental Material

## Data Availability

The data that support the findings of this study are available on request from the corresponding author.
