# Supplementary material for: Specific alterations in gut microbiota in patients with chronic kidney disease: an updated systematic review
Source: Ren Fail. 2021 Jan 6;43(1):102–12. doi: 10.1080/0886022X.2020.1864404 (PMC7808321; doi:10.1080/0886022X.2020.1864404)
Supplement: Supplemental Material [file IRNF_A_1864404_SM8300.docx]

**Specific alterations of gut microbiota in patients with chronic kidney disease: an updated systematic review**

Jin Zhao , Xiaoxuan Ning, Baojian Liu, Ruijuan Dong, Ming Bai and Shiren Sun

**Table S 1** Search strategy

**Table S 2** Quality assessment of selected studies using the Newcastle-Ottawa Scale.

**Table S 3** Details of gene analysis methods and fecal sample collection.

**Table S 4** Alpha diversity between case groups and control groups.

**Table S 5** Beta diversity between case groups and control groups.

**Table S 6** Alteration of gut microbiota at the phylum level.

**Table S 7** Alteration of gut microbiota at the class level.

**Table S 8** Alteration of gut microbiota at the order level.

**Table S 9** Alteration of gut microbiota at the family level.

**Table S 10** Alteration of gut microbiota at the genus level.

**Table S 11** Alteration of gut microbiota at the species level.

**Table S 12** Metabolites derived from gut microbiota.

**Table S 13** Comparison of parameters of gut permeability.

**Table S 1. Search strategy**

#1 MeSH Renal insufficiencies,Chronic

#2 Chronic Renal Insufficiencies

#3 Renal Insufficiencies, Chronic

#4 Chronic Renal Insufficiency

#5 Kidney Insufficiency, Chronic

#6 Chronic Kidney Insufficiency

#7 Chronic Kidney Insufficiencies

#8 Kidney Insufficiencies, Chronic

#9 Chronic Kidney Diseases

#10 Chronic Kidney Disease

#11 Disease, Chronic Kidney

#12 Diseases, Chronic Kidney

#13 Kidney Disease, Chronic

#14 Kidney Diseases, Chronic

#15 Chronic Renal Diseases

#16 Chronic Renal Disease

#17 Disease, Chronic Renal

#18 Diseases, Chronic Renal

#19 Renal Disease, Chronic

#20 Renal Diseases, Chronic

#21 “CKD”

#22 or/1-21

#23 MeSH Gastrointestinal Microbio*

#24 gut microbiota*

#25 fecal microbio*

#26 faecal microbio*

#27 intestinal microbio*

#28 or/23-27

#29 MeSH cross-sectional study

#30 MeSH case-control study

#31 #29 OR #30

#32 22and 28 and 31

****Table S 2. Quality assessment of selected studies using the Newcastle-Ottawa Scale**.**

| **Study** | **Selection** | | | | | **Comparability** | | **Ascertainment exposure** | | | **Score** |
| --- | --- | --- | --- | --- | --- | --- | --- | --- | --- | --- | --- |
|  | **1** | **2** | **3** | | **4** | **5A** | **5B** | **6** | **7** | **8** |  |
| Feiqian Wang et al.2012 | ***** | ***** | ***** | ***** | | ***** |  | ***** | ***** |  | 7***** |
| I-Kuan Wang et al.2012 | ***** | ***** |  | ***** | | ***** |  | ***** | ***** |  | 6***** |
| Nosratola D. Vaziri et al.2013 | ***** | ***** |  | ***** | | ***** |  | ***** | ***** |  | 6***** |
| Shuanghong,Jiang et al.2017 | ***** | ***** |  | ***** | | ***** |  | ***** | ***** |  | 6***** |
| Stadlbauer, Vanessa et al.2017 | ***** | ***** |  | ***** | | ***** |  | ***** | ***** |  | 6***** |
| Guirong YE et al.2018 | ***** | ***** |  | ***** | | ***** |  | ***** | ***** |  | 6***** |
| Yang Li et al.2019 | ***** | ***** |  | ***** | | ***** |  | ***** | ***** |  | 6***** |
| Terpstra, M. L. 2019 | ***** | ***** |  | ***** | | ***** |  | ***** | ***** |  | 6***** |
| Xifan Wang et al.2020 | ***** | ***** |  | ***** | | ***** |  | ***** | ***** |  | 6***** |
| Amanda F Barros et al 2015 | ***** | ***** |  | ***** | | ***** |  | ***** | ***** |  | 6***** |
| Elisabetta Margiotta et al 2020 | ***** | ***** |  | ***** | |  |  | ***** | ***** |  | 5***** |
| Kaiyu Xu et al.2017 | ***** | ***** |  | ***** | | ***** |  | ***** | ***** |  | 6***** |
| Salguero, Maria V et al.2019 | ***** | ***** |  | ***** | | ***** |  | ***** | ***** |  | 6***** |
| Al-Obaide, M. A. I. et al.2017 | ***** | ***** |  | ***** | | ***** |  | ***** | ***** |  | 6***** |
| FengXia Li et al.2019 | ***** | ***** |  | ***** | | ***** |  | ***** | ***** |  | 6***** |
| Shuanghong,Jiang et al.2016 | ***** | ***** |  | ***** | | ***** |  | ***** | ***** |  | 6***** |
| HengzhongLun et al.2018 | ***** | ***** |  | ***** | | ***** |  | ***** | ***** |  | 6***** |
| Wang, Siqi et al.2019 | ***** | ***** |  | ***** | | ***** |  | ***** | ***** |  | 6***** |
| I-Wen Wu et al.2020 | ***** | ***** |  | ***** | | ***** |  | ***** | ***** |  | 6***** |
| Xiaofang Hu et al. 2020 | ***** | ***** |  | ***** | | ***** |  | ***** | ***** |  | 6***** |
| Zhigang Ren et al. 2020 | ***** | ***** |  | ***** | | ***** |  | ***** | ***** |  | 6***** |
| Maria De Angelis et al.2014 | ***** | ***** |  | ***** | | ***** |  | ***** | ***** |  | 6***** |
| Xiaofang Hu et al. 2020 | ***** | ***** |  | ***** | | ***** |  | ***** | ***** |  | 6***** |
| ZhengXia Zhong et al.2020 | ***** | ***** |  | ***** | | ***** |  | ***** | ***** |  | 6***** |
| Sibei Tao et al.2019 | ***** | ***** | ***** | ***** | | ***** |  | ***** | ***** |  | 7***** |

| ****Table S 3. Details of gene analysis methods and fecal sample collection.**** | | | | | |
| --- | --- | --- | --- | --- | --- |
| **Study** | **Analysis methods** | **Samples** | **Sampling container** | **Manufacturer** | **Specimens protection** |
| Feiqian Wang et al.2012 | PCR and pyrosequencing | serum/stool | N/A | N/A | N/A |
| I-Kuan Wang et al.2012 | Real-time PCR analysis | stool | N/A | N/A | N/A |
| Nosratola D. Vaziri et al.2013 | 16S rRNA gene PhyloChip analysis | stool | PSP Spin Stool DNA PLUS Kit | Invitek Biotechnology and Biodesign, Berlin- Buch, Germany | DNA stabilization, isolation, and purification components |
| Shuanghong,Jiang et al.2017 | qRT-PCR and 16S rRNA gene sequence | stool | TIANamp Stool DNA Kit | TIANGEN Biotech, Beijing, China | N/A |
| Stadlbauer, Vanessa et al.2017 | 16S rRNA gene sequence | stool | N/A | N/A | N/A |
| Guirong Ye et al.2018 | 16S rRNA gene sequence | stool | N/A | N/A | N/A |
| Yang Li et al 2019 | 16S rRNA gene sequence, | stool | Sterilized 2-ml tubes | N/A | Pure ethanol |
| Terpstra, M. L. 2019 | qPCR | stool | N/A | N/A | N/A |
| Xifan Wang et al.2020 | Metagenome shotgun sequencing | serum/stool | N/A | N/A | N/A |
| Kaiyu Xu et al.2017 | 16S rRNA gene sequence | stool | N/A | N/A | N/A |
| Salguero, Maria V et al.2019 | 16S rRNA gene sequence | stool | N/A | N/A | N/A |
| Al-Obaide, M. A. I. et al.2017 | 16S rRNA gene sequence | stool | N/A | N/A | N/A |
| Amanda F Barros et al. 2015 | Denaturing gradient gel electrophoresis | stool | Plastic bottle | N/A | N/A |
| Elisabetta Margiotta et al. 2020 | 16S rRNA gene sequence | stool | N/A | N/A | N/A |
| FengXia Li et al. 2019 | 16S rRNA gene sequence | stool | N/A | N/A | N/A |
| Shuanghong,Jiang et al.2016 | Quantitative real-time PCR | stool | N/A | N/A | N/A |
| HengzhongLun et al.2018 | 16S rRNA gene sequence | stool | Sterile plastic cup | N/A | N/A |
| Wang, Siqi et al.2019 | qRT-PCR | serum/stool | N/A | N/A | N/A |
| I-Wen Wu et al. 2020 | 16S rRNA gene sequence | stool | N/A | N/A | N/A |
| Xiaofang Hu et al. 2020 | 16S ribosomal DNA sequencing | stool | N/A | N/A | N/A |
| Zhigang Ren et al.2020 | 16S ribosomal DNA sequencing | stool | N/A | N/A | N/A |
| Maria De Angelis et al 2014 | Bacterial culture; Pyrosequencing analysis of 16S rRNA gene | stool | Sterile plastic box | Sigma-Aldrich, St. Louis,  MO, USA | RNA later |
| Xiaofang Hu et al. 2020 | 16S rRNA gene sequence | stool | N/A | N/A | N/A |
| ZhengXia Zhong et al.2020 | 16S rRNA gene sequence | stool | N/A | N/A | N/A |
| Sibei Tao et al 2019 | 16S rRNA gene sequence | stool | N/A | N/A | N/A |

PCR, polymerase chain reaction; qRT-PCR:quantitative real-time polymerase chain reaction.

****Table S 4. Alpha diversity between case groups and control groups.****

| **Study** | **Results description** | **Alpha-diversity Index** | **Conclusion** |
| --- | --- | --- | --- |
| Feiqian Wang et al.2012 | N/A | N/A | N/A |
| I-Kuan Wang et al. 2012 | N/A | N/A | N/A |
| Nosratola D. Vaziri et al.2013 | Mean relative richness (summarized at subphylum) for ESRD and control groups was similar. | observed species | similar |
| Shuanghong,Jiang et al.2017 | Diversity was similar between healthy controls and ESRD patients. | Chao1 | similar |
| Stadlbauer, Vanessa et al.2017 | Diversity was significantly lower in HD and PD patients | Chao1 | decrease |
| Guirong YE et al.2018 | RT recipients had the lowest microbial richness followed by CKD,and the highest microbial richness occurred in the controls. | Chao1 | decrease |
| Yang Li et al 2019 | Microbial diversity and abundance were higher in the healthy control group than in both CKD 5 groups | Shannon;ACE | decrease |
| Terpstra, M. L. et al 2019 | N/A | N/A | N/A |
| Xifan Wang et al. 2020 | Microbial diversity of the ESRD microbiome markedly differed from that of healthy controls. | Shannon | decrease |
| Amanda F Barros et al 2015 | N/A | N/A | N/A |
| Elisabetta Margiotta et al 2020 | No differences in alpha diversity between CKD and healthy controls | Chao1, Shannon | similar |
| Kaiyu Xu et al.2017 | Both of these indices were significantly lower in the CKD group (P < 0.001). | Shannon;PD-whole tree | decrease |
| Salguero,Maria V et al.2019 | N/A | N/A | N/A |
| Al-Obaide, M. A. I. et al.2017 | N/A | N/A | N/A |
| FengXia Li et al 2019 | Alpha diversity was markedly, but not significantly, lower in the fecal microbiota obtained from patients with CKD than in those from the HC subjects. | Shannon and Simpson | similar |
| Shuanghong,Jiang et al.2016 | N/A | N/A | N/A |
| HengzhongLun et al.2018 | N/A | N/A | N/A |
| Wang, Siqi et al.2019 | N/A | N/A | N/A |
| I-Wen Wu et al. 2020 | Significant differences in bacterial species richness and evenness were detected among different CKD stages, and between moderate CKD and HC subjects. | Chao 1 | decrease |
| Xiaofang Hu et al. 2020 | Differences were observed in the microbial diversity of fecal samples from CKD patients and healthy controls. | Chao1, | decrease |
| Zhigang Ren et al.2020 | Gut microbial diversity was significantly reduced in CKD compared to the healthy controls. | Shannon; Chao; ACE | decrease |
| Maria De Angelis et al.2014 | Alpha diversity was decreased in IgAN patients compared with healthy controls. | Chao1, Shannon, Good’s coverage | decrease |
| Xiaofang Hu et al. 2020 | Alpha diversity were significantly decreased in the IgAN patients compared to those in the healthy controls | Chao, ACE, observed species diversity | decrease |
| ZhengXia Zhong et al.2020 | Alpha diversity analysis showed nosignificant differences in taxon richness and evenness | Chao; ACE; Sobs | similar |
| Sibei Tao et al.2019 | Different richness of OTUs were found in DM and DN patients | Sobs | N/A |

PD-whole tree, phylogenetic diversity whole tree; ESRD,end stage renal disease; HD, hemodialysis; PD,peritoneal dialysis; CKD,chronic kidney disease; RT, renal transplantation; N/A, not available; OTU, operational taxonomic unit.

****Table S 5. Beta diversity between case groups and healthy controls.****

| **Study** | **Results description** | **Beta-diversity Index** | **Conclusion** |
| --- | --- | --- | --- |
| Feiqian Wang et al.2012 | N/A | N/A | N/A |
| I-Kuan Wang et al.2012 | N/A | N/A | N/A |
| Nosratola D. Vaziri et al.2013 | Tighter clustering shown in the control group than in the ESRD group | NMDS | distinct |
| Shuanghong,Jiang et al.2017 | No separation trend of healthy controls and ESRD patients. | PCoA | similar |
| Stadlbauer, Vanessa et al.2017 | Differences between controls and HD or PD patients was significant. | Redundancy analysis | distinct |
| Guirong Ye et al.2018 | Gut microbiota in RT recipients and CKD patients could be separated from the controls. | PCA | distinct |
| Yang Li et al.2019 | Healthy control group was significantly different from the CKD5 group. | PCoA | distinct |
| Terpstra, M. L. et al.2019 | N/A | N/A | N/A |
| Xifan Wang et al.2020 | Taxonomic composition of the ESRD microbiome markedly differed from that of healthy controls | NMDS | distinct |
| Amanda F Barros et al.2015 | N/A | N/A | N/A |
| Elisabetta Margiotta et al.2020 | No significant differences in microbial composition were found between CKD and patients. | PCA | similiar |
| Kaiyu Xu et al.2017 | According to the unweighted UniFrac distance analysis, the distance between the CKD group and the control group was significantly different (ADONIS analysis, P < 0.001, R2 = 0.071) | PCoA | distinct |
| Salguero, Maria V et al.2019 | N/A | N/A | N/A |
| Al-Obaide, M. A. I.et al.2017 | N/A | N/A | N/A |
| FengXia Li et al.2019 | The beta diversity of the fecal microbiota was significantly reduced in the CKD compared with the healthy control group. | PCoA | distinct |
| Shuanghong Jiang et al.2016 | N/A | N/A | N/A |
| HengzhongLun et al.2018 | Gut microbiota of patients with CKD were distinct from those of healthy controls | PCA; NMDS | distinct |
| Wang, Siqi et al.2019 | N/A | N/A | N/A |
| I-Wen Wu et al.2020 | Notable discrimination among different stages of CKD and non-CKD controls | Bray-Curtis similarity index | distinct |
| Xiaofang Hu et al. 2020 | Differences were observed in the microbial composition of fecal samples from CKD patients and healthy controls. | N/A | distinct |
| Zhigang Ren et al.2020 | The PCoA and the NMDS based on OTUs distribution showed that the gut taxonomic composition was significantly different between CKD. | PCoA; NMDS | distinct |
| Maria De Angelis et al.2014 | Fecal samples of IgAN patients and healthy controls were clustered in different zones of the 3-D plot. | PCA | distinct |
| Xiaofang Hu et al.2020 | A separation trend in the β diversity between the IgAN patients and healthy controls | PCoA | distinct |
| ZhengXia Zhong et al.2020 | There was a clear separation in the composition of the gut microbiota between the patients with IgAN and HCs. | PCoA | distinct |
| Sibei Tao et al.2019 | Microbiota composition of healthy controls, DM, and DN patients were significantly different. | PCoA | distinct |

ESRD,end stage renal disease; HD, hemodialysis; PD,peritoneal dialysis; CKD,chronic kidney disease; RT, renal transplantation; DM, type 2 diabetes mellitus; DN diabetic nephropathy; PcoA,principal coordinate analysis; PCA, principal Component analysis; NMDS,Non-metric multidimensional scaling; N/A, not available; HC,Healthy controls.

****Table S 6. Alteration of gut microbiota at phylum level.****

| **Study** | **Higher abundant phylotypes** | | **Lower abundant phylotypes** |
| --- | --- | --- | --- |
| Feiqian Wang et al.2012 | N/A | | N/A |
| I-Kuan Wang et al.2012 | N/A | | N/A |
| Nosratola D. Vaziri et al.2013 | N/A | | N/A |
| Shuanghong,Jiang et al.2017 | N/A | | N/A |
| Stadlbauer, Vanessa et al.2017 | N/A | N/A | |
| Guirong Ye et al.2018 | Proteobacteria and Bacteroidetes | | Firmicutes |
| Yang Li et al.2019 | NH：Firmicutes, Actinobacteria, Proteobacteria,Fusobacteria  HD：Firmicutes, Actinobacteria, Fusobacteria, | | Bacteroidetes |
| Terpstra, M. L. et al.2019 | N/A | | N/A |
| Xifan Wang et al.2020 | N/A | | N/A |
| Amanda F Barros et al.2015 | N/A | | N/A |
| Elisabetta Margiotta et al.2020 | N/A | | N/A |
| Kaiyu Xu et al.2017 | Proteobacteria | | Firmicutes and Actinobacteria |
| Salguero, Maria V et al.2019 | Proteobacteria,  Verrucomicrobia and Fusobacteria | |  |
| Al-Obaide, M. A. I.et al | N/A | | N/A |
| FengXia Li et al.2019 | Actinobacteria | | Verrucomicrobia |
| Shuanghong Jiang et al.2016 | N/A | | N/A |
| HengzhongLun et al.2018 | Proteobacteria and Bacteroidetes | | Firmicutes |
| Wang, Siqi et al.2019 | N/A | | N/A |
| I-Wen Wu et al.2020 | N/A | | N/A |
| Xiaofang Hu et al. 2020 | Proteobacteria | | Synergistetes |
| Zhigang Ren et al. 2020 | Proteobacteria, Actinobacteria, Fusobacteria, Lentisphaerae, and Candidate_division_TM7 | | Firmicutes and Verrucomicrobia  Bacteria unclassified and Cyanobacteria |
| Maria De Angelis et al.2014 | Firmicutes | | Bacteroidetes |
| Xiaofang Hu et al.2020 | Fusobacteria | | Synergistetes |
| ZhengXia Zhong et al.2020 | Bacteroidetes | | Firmicutes, Actinobacteria, and Tenericutes |
| Sibei Tao et al.2019 | Proteobacteria | | N/A |

HD, hemodialysis; PD,peritoneal dialysis; N/A, not available. NH: no– hemodialysis

****Table S 7. Alteration of gut microbiota at the class level.****

| **Study** | **Higher abundant phylotypes** | **Lower abundant phylotypes** |
| --- | --- | --- |
| Feiqian Wang et al.2012 | N/A | N/A |
| I-Kuan Wang et al.2012 | N/A | N/A |
| Nosratola D. Vaziri et al.2013 | N/A | N/A |
| Shuanghong,Jiang et al.2017 | Deltaproteobacteria | N/A |
| Stadlbauer, Vanessa et al.2017 | HD:Bacilli, Epsilonproteobacteria and Gammaproteobacteria;  PD:Alphaproteobacteria | Erysipelotrichia |
| Guirong YE et al.2018 | Deltaproteobacteria | Clostridia, Betaproteobacteria, Mollicutes |
| Yang Li et al 2019 | NH: Clostridia, Bacilli, Actinobacteria, Negativicutes, Coriobacteriia, Fusobacteriia,  HD: Clostridia, Actinobacteria, Erysipelotrichia, Negativicutes, Coriobacteriia, Alphaproteobacteria, Fusobacteria. | Bacteroidia, |
| Terpstra, M. L. 2019 | N/A | N/A |
| Xifan Wang et al. 2020 | N/A | N/A |
| Amanda F Barros et al 2015 | N/A | N/A |
| Elisabetta Margiotta et al 2020 | N/A | N/A |
| Kaiyu Xu et al.2017 | N/A | N/A |
| Salguero,Maria V et al.2019 | N/A | N/A |
| Al-Obaide, M. A. I. et al.2017 | N/A | N/A |
| FengXia Li et al 2019 | Actinobacteria | Verrucomicrobia, Bacilli, Betaproteobacteria, Synergistia, Epsilonproteobacreria, Deferribacteres, |
| Shuanghong,Jiang et al.2016 | N/A | N/A |
| HengzhongLun et al.2018 | Bacteroidia and Gammaproteobacteria | Clostridia |
| Wang, Siqi et al.2019 | N/A | N/A |
| I-Wen Wu et al. 2020 | N/A | N/A |
| Xiaofang Hu et al. 2020 |  |  |
| Zhigang Ren et al. 2020 | Actinobacteria, Gammaproteobacteria, Deltaproteobacteria Alphaproteobacteria, Bacilli, Fusobacteria, Erysipelotrichia,  and Lentisphaeria. | Clostridia, Verrucomicrobia, Cyanobacteria, Betaproteobacteria, and Firmicutes_unclassified. |
| Maria De Angelis et al 2014 | N/A | N/A |
| Xiaofang Hu et al. 2020 | N/A | N/A |
| ZhengXia Zhong et al.2020 | Bacteroidia, Fusobacteria, Gammaproteobacteria | Clostridia, Actinobacteria |
| Sibei Tao, et al 2019 | N/A | N/A |

HD, hemodialysis; PD,peritoneal dialysis; NH, no haemodialysis

****Table S 8. Alteration of gut microbiota at the order level.****

| **Study** | **Higher abundant phylotypes** | **Lower abundant phylotypes** |
| --- | --- | --- |
| Feiqian Wang et al.2012 | N/A | N/A |
| I-Kuan Wang et al.2012 | N/A | N/A |
| Nosratola D. Vaziri et al.2013 | N/A | N/A |
| Shuanghong,Jiang et al.2017 | Desulfovibrionales | Pseudomonadales, Pasteurellales |
| Stadlbauer, Vanessa et al.2017 | Abundance of Lactobacillales, Campylobacterales, Enterobacteriales, Erysipelotrichales and Gastranaerophilales were significantly changed. | |
| Guirong YE et al.2018 | Desulfovibrionales | Bifidobacteriales, Clostridiales, Burkholderiales, RF39 |
| Yang Li et al 2019 | NH: Clostridiales, Erysipelotrichia, Bifidobacteriales, Lactobacillales, Fusobacteriales, Burkholderiales, Pasteurellales, Neisseriales, Verrucomicrobiales,  HD:Clostridiales, Lactobacillales, Erysipelotrichaceae, Bifidobacteriales, Selenomonadales, Coriobacteriales, Caulobacterales, Burkholderiales, Fusobacteriales, Neisseriales, Pasteurellales | Bacteroidales, Enterobacteriales, |
| Terpstra, M. L. 2019 | N/A | N/A |
| Xifan Wang et al. 2020 | N/A | N/A |
| Amanda F Barros et al 2015 | N/A | N/A |
| Elisabetta Margiotta et al 2020 | N/A | N/A |
| Kaiyu Xu et al.2017 | Enterobacteriales | Actinomycetales, Clostridiales, Caulobacterales, Rhizobiales, Rickettsiales, and Burkholderiales |
| Salguero,Maria V et al.2019 | N/A | N/A |
| Al-Obaide, M. A. I. et al.2017 | N/A | N/A |
| FengXia Li et al 2019 | Actinomycetales, Coriobacteriales | Deferribacterales, Lactobacillales, Burkholderiales, Campylobacterales, Synergistales, and Verrucomicrobiales. |
| Shuanghong,Jiang et al.2016 | N/A | N/A |
| HengzhongLun et al.2018 | Enterobacteriales | N/A |
| Wang, Siqi et al.2019 | N/A | N/A |
| I-Wen Wu et al. 2020 | N/A | N/A |
| Xiaofang Hu et al. 2020 | N/A | N/A |
| Zhigang Ren et al. 2020 | Lactobacillales, Enterobacteriales, Erysipelotrichales, Desulfovibrionales, Coriobacteriales, Fusobacteriales, Pasteurellales, Rhodospirillales, and Victivallales. | Clostridiales, Burkholderiales, Verrucomicrobiales Firmicutes_unclassified, and Pseudomonadales |
| Maria De Angelis et al 2014 | N/A | N/A |
| Xiaofang Hu et al. 2020 | N/A | N/A |
| ZhengXia Zhong et al.2020 | Bacteroidales, Fusobacteriales, Pasteurellales | Clostridiales, Coriobacteriales, Bifidobacteriales, and Anaerolineales |
| Sibei Tao et al.2019 | Coriobacteriales | N/A |

Abbreviation: HD, hemodialysis; PD,peritoneal dialysis; N/A, not available. NH, no hemodialysis.

****Table S 9. Alteration of gut microbiota at the family level.****

| **Study** | **Higher abundant phylotypes** | | **Lower abundant phylotypes** |
| --- | --- | --- | --- |
| Feiqian Wang et al.2012 | N/A | | N/A |
| I-Kuan Wang et al.2012 | N/A | | N/A |
| Nosratola D. Vaziri et al.2013 | Nesterenkonia,Catabacter,Peptostreptococcaceae, Polyangiaceae, Alteromonas, Enterobacteriaceae, Halomonadaceae, Methylococcaceae, Moraxellaceae, Pseudomonadaceae, and Thiothrix | | N/A |
| Shuanghong,Jiang et al.2017 | Bacteroidaceae, Desulfovibrionaceae | | Pseudomonadaceae, Pasteurellaceae, Prevotellaceae, and Alcaligenaceae. |
| Stadlbauer, Vanessa et al.2017 | Comamonadaceae, Campylobacteraceae, Streptococcaceae, Enterobacteriaceae, Bacteroidales S24-7 group,Rhodospirillaceae, Erysipelotrichaceae were differentially abundant. | | |
| Guirong YE et al.2018 | Odoribacteraceae and Desulfovibrionaceae | | Bifidobacteriaceae, Prevotellaceae, Paraprevotellaceae, Clostridiaceae, Ruminococcaceae, Veillonellaceae, Alcaligenaceae, and RF39_f_. |
| Yang Li et al 2019 | NH:Lachnospiraceae, Erysipelotrichaceae, Bifidobacteriaceae, Streptococcaceae, Coriobacteriaceae, Fusobacteriaceae, Alcaligenaceae, Pasteurellaceae, Neisseriaceae, Verrucomicrobiaceae, Enterococcaceae  HD: Lachnospiraceae, Streptococcaceae, ErysipelotrichaceaeBifidobacteriaceae, Enterococcaceae, Acidaminococcaceae, Coriobacteriaceae, Caulobacteraceae, Alcaligenaceae, Neisseriaceae, Pasteurellaceae, Fusobacteriaceae. | | Bacteroidaceae, Prevotellaceae, Ruminococcaceae, Veillonellaceae, Christensenellaceae, Prophyromonadaceae, Rikenellaceae,Anaplasmataceae, Colwelliaceae, BacteroidalesS24-7group. |
| Terpstra, M. L. 2019 | N/A | | N/A |
| Xifan Wang et al. 2020 | N/A | | N/A |
| Amanda F Barros et al 2015 | N/A | | N/A |
| Elisabetta Margiotta et al.2020 | No significant differences were found between the two groups at the family level | | |
| Kaiyu Xu et al.2017 | Corynebacteriaceae and Enterobacteriaceae | Intrasporangiaceae, Mycobacteriaceae, Prevotellaceae, Rikenellaceae, Lachnospiraceae, Caulobateraceae, Bradyrhizobiaceae, Alcaligenaceae, Aeromonadaceae, Mitochondria,Ruminococcaceae. | |
| Salguero,Maria V et al.2019 | N/A | N/A | |
| Al-Obaide, M. A. I. et al.2017 | N/A | N/A | |
| FengXia Li et al 2019 | Coriobacteriaceae, Clostridiaceae1,Methylobacteriaceae | Micrococcaceae, Deferribacteraceae, Lactobacillaceae, Eubacteriaceae, Sutterellaceae, Helicobacteraceae, Synergistaceae, Verrucomicrobiaceae. | |
| Shuanghong,Jiang et al.2016 | N/A | N/A | |
| HengzhongLun et al.2018 | Bacteroidaceae and Enterobacteriaceae | Prevotellaceae,Veillonellaceae, and Lachnospiraceae | |
| Wang, Siqi et al.2019 | N/A | N/A | |
| I-Wen Wu et al.2020 | N/A | Lachnospiraceae | |
| Xiaofang Hu et al. 2020 | N/A | N/A | |
| Zhigang Ren et al. 2020 | Enterobacteriaceae, Rikenellaceae, Porphyromonadaceae, Acidaminococcaceae,  Christensenellaceae, Erysipelotrichaceae, Desulfovibrionaceae, Streptococcaceae,  Bacteroidales_unclassified, Coriobacteriaceae, Fusobacteriaceae, Pasteurellaceae, Family_XIII, Rhodospirillaceae, VadinBB60, Victivallaceae. | Lachnospiraceae, Alcaligenaceae, and Moraxellaceae, Clostridiales_unclassified,  Verrucomicrobiaceae, Firmicutes_unclassified, Enterococcaceae, Defluviitaleaceae | |
| Maria De Angelis et al 2014 | Streptococcaceae, Eubacteriaceae, Alcaligenaceae, Enterobacteriaceae and Coriobacteriaceae | Lactobacillaceae, Bacteroidaceae, Bifidobacteriaceae and Prevotellaceae | |
| Xiaofang Hu et al. 2020 | Enterobacteriaceae | Synergistaceae | |
| ZhengXia Zhong et al.2020 | Bacteroidaceae, Fusobacteriaceae, Pasteurellaceae, Tannerellaceae, and Carnobacteriaceae | Coriobacteriaceae; Bifidobacteriaceae; Peptostreptococcaceae; Anaerolineaceae; Eggerthellaceae;Norank_O_Mollicutes_RF39 | |
| Sibei Tao et al.2019 | Coriobacteriaceae | Prevotellaceae | |

PD, peritoneal dialysis; N/A, not available.

****Table S 10. Alteration of gut microbiota at the genus level.****

| **Study** | **Higher abundant phylotypes** | **Lower abundant phylotypes** |
| --- | --- | --- |
| Feiqian Wang et al.2012 | N/A | N/A |
| I-Kuan Wang et al.2012 | N/A | N/A |
| Nosratola D. Vaziri et al.2013 | Brachybacterium, Catenibacterium. | N/A |
| Shuanghong,Jiang et al.2017 | Bacteroides,Parabacteroides,Escherichia_Shigella,Subdoligranulum,Fusobacterium,Parasporobacterium,and Paraprevotella. | Prevotella, Roseburia, Faecalibacterium,Caternibacterium, Dorea, Dialister,Slackia, Megamonas, Alcaligenaceae, Pseudomonas,Coprococcus, Clostridium, Anaerobacter, Oribacterium and Parasutterella. |
| Stadlbauer, Vanessa et al.2017 | **PD:**Talassospira, Eisenbergiella, Ruminococcaceae. **HD**:Escherichia_Shigella, Streptococcus, Enterobacter, and Blautia. | Coprococcus, Holdemanella, Asteroleplasma, Paraprevotella, Prevotella, Romboutsia, LachnospiraceaUCG008, and Pelomonas, |
| Guirong Ye et al.2018 | Odoribacter, Dorea, Oscillospira, Ruminococcus, Phascolarctobacterium, Bilophila, and Desulfovibrio. | Bifidobacterium, Prevotella, Clostridiaceae_g, Clostidium, Anaerostipes, Butyrivibrio, Coprococcus, Lachnospira, Lachnospiraceae_g, Roseburia,Shuttleworthia, Faecalibacterium, Ruminococcaceae_g, Dialister, Megamonas, Veillonella,andSutterella. |
| Yang Li et al.2019 | **NH-HC:** Bifidobacterium, Subdoligrannulum, Streptococcus,  ErysipelotrichaceaeUCG_003,Erysipelatoclostridium, collinsella, ruminococcaceaeUCG_014, Ruminococcus2;Klebsiella, Eubacteriumrectalegroup, Roseburia, Fusobacterium,Paresutterella, Haemophilus, RuminococcaceaeUCG_013, Faecalitalea, Ruminiclostridium5, Neisseria, RuminococcaceaeUCG_011, Akkermansia, Shuttleworthia, Dialister, ErysipelotrichaceaeUCG_006.  **HD-HC:** Streptococcus, Bifidobacterium, Blautia, Subdoligranulum, Lachnoclostridium, Phascolarctobacterium, ErysipelotrichaceaeUCG_003, Ruminococcustorquesgroup, Erysipelatoclostridium, LachnospiraceaeUCG_008, Ruminococcus2, Collinsella, Holdemanella, Tyzzerella4, Agromyces, Herbaspirillum, Howardella, Veillonella, Neisseria, Asticcacaulis, Parasutterella,Haemophilus, Lactobacillus,Dialister, Niabella, Fusobacterium,Dorea, | **NH-HC:**Bacteroides, Faecalibacterium, Prevotella9, Escherichia_shigella, Enterobacter, Megamonas, Prevotella2, ChristensenellaceaeR_Group, Parabacteroides, Alistipes, Serratia, PrevotellaceaeNK3B31group,RuminococcaceaeUCG_002, Eubacteriumventriosumgroup, Enterorhabdus, Flavobacterium, LachnospiraceaeUCG_004.  **HD-HC:**Bacteroides, Faecalibacterium, Prevotella9, Megamonas, ChristensenellaceaeR_Group, Prevotella2, Enterobacter, RuminococcaceaeUCG_002, Alistipes, Parabacteroides, Tyzzerella3, Eubacteriumventriosumgroup, Roseburia, LachnospiraceaeUCG_004. |
| Terpstra, M. L. et al.2019 | N/A | N/A |
| Xifan Wang et al.2020 | N/A | N/A |
| Amanda F Barros et al.2015 | N/A | N/A |
| Elisabetta Margiotta et al.2020 | Lactobacillus, Coprobacillus, Anaerotruncus, Citrobacter and Ruminococcus torques | Prevotella spp., F. prausnitzii and Roseburia spp |
| Kaiyu Xu et al.2017 | Rothia, Enterococcus, Clostridium, Desulfotomaculum | KnoelliaMycobaterium, Atopobium, Prevotella, Coprococcus, Dorea, Roseburia, Acidaminococcus, Megamonas, Sutterella, Acidovorax, Enterobacter and Synergistes. |
| Salguero, Maria V et al.2019 | N/A | N/A |
| Al-Obaide, M. A. I.et al.2017 | Anaerococcus, Clostridium, Desulfitobacter, Enterococcus, Streptococcus, Desulfovibrio, Enterobacter, Escherichia, Klebsiella, Proteus, Pseudomonas, Acinetobacter, and Citrobacter | Bifidobacterium |
| FengXia Li et al.2019 | Methylobacterium, Clostridium sensusstricto, Desulfovibrio, Paraprevitella, Alloprevotella,Clostridium IV. | Akkermansia, Parasutteralla, Lactobacillus, Olsenella, Leuconostoc, Pyramidobacter, Synergistes, Parvibacter, Helicobacter, Mucispirillum, Lactococcus, Enterorhadbus, Allobaculum, Rothia, Eubacterium, Oribacterium. |
| Shuanghong Jiang et al.2016 | N/A | N/A |
| HengzhongLun et al.2018 | Bacteroides and Escherichia_Shigella | Roseburia |
| Wang, Siqi et al.2019 | N/A | N/A |
| I-Wen Wu et al.2020 | Bacteroides, Blautia, Escherichia-Shigella, Collinsella, Lachnoclostridium, and Lactobacillus. | Paraprevotella, Tyzzerella 3, Lachnospiraceae ND3007 group, Eubacterium ruminantium group, Pseudobutyrivibrio, Dialister and Roseburia. |
| Xiaofang Hu et al. 2020 | Escherichia-Shigella, Parabacteroides, Flavonifractor, and Pseudomonas. | Pyramidobacter and Prevotellaceae_UCG-001 |
| Zhigang Ren et al. 2020 | Escherichia_Shigella, Subdoligranulum, Alistipes, Parabacterorides, Klebsiella, Phascolarctobacterium, Citrobacter, Akkermansia, Streptococcus, Barnesiella, Norank, Veillonella, Desulfovibrio, Bilophila, Anaerotruncus, Flavonifractor, Fusobacterium, Collinsella, Odoribacter, Haemophilus, Holdemania, Christensenella, Granulicatella. | Faecalibacterium, Lncertae_Sedis, Blautia, Pseudobutyrivibrio, Roseburia, Lachnospira, Anaerostipes, Parasutterella, Sphingomonas. |
| Maria De Angelis et al.2014 | N/A | N/A |
| Xiaofang Hu et al.2020 | Escherichia-Shigella, Hungatella, and Eggerthella. | Rectale_group, Barnesiella, Ruminococcaceae_NK4A214_group, Prevotellaceae_NK3B31_group, Prevotellaceae_UCG-001, Coprococcus_2, Lachnospiraceae_FCS020_group, and Pyramidobacter. |
| ZhengXia Zhong et al.2020 | Bacteroides, Escherichia-Shigella, and Lachnoclostridium | Subdoligranula, Blautia, Subdoligranulum, Prevotella_9, Eubacterium hallii, and Bifidobacterium. |
| Sibei Tao et al.2019 | Hungatella, Caproiciproducens, Family_XIII_AD3011_group, Holdemanella, Bilophila and Escherichia-Shigella | Prevotella_9 |

PD, peritoneal dialysis; HD, haemodialysis. NH, non-hemodialysis; HD-HC, hemodialysis versus healthy control;NH-HC**,** no-hemodialysis versus healthy control.

****Table S 11. Alteration of gut microbiota at the species level.****

| **Study** | **Higher abundant phylotypes** | | **Lower abundant phylotypes** |
| --- | --- | --- | --- |
| Feiqian Wang et al.2012 | Klebsiella spp, Proteus spp, Escherichia spp, Enterobacter spp, and Pseudomonas spp. | | N/A |
| I-Kuan Wang et al.2012 | N/A | | All Bifidobacteria, Bifidobacterium catenulatum, B. longum, B. bifidum, Lactobacillus plantarum, L. paracasei, and Klebsiella pneumoniae. |
| Nosratola D. Vaziri et al.2013 | N/A | | N/A |
| Shuanghong,Jiang et al.2017 | N/A | | N/A |
| Stadlbauer, Vanessa et al.2017 | N/A | | N/A |
| Guirong YE et al.2018 | N/A | | N/A |
| Yang Li et al 2019 | N/A | | N/A |
| Terpstra, M. L. 2019 | Patients with ESRD did not have a significantly decreased amount F. prauznitzii, E. rectale and Roseburia spp. or the BCoAT gene. | | |
| Xifan Wang et al.2020 | Anaerostipes caccae, Eggerthella lenta, Flavonifractor spp (mainly F. plautii), Intestinimonas, Alistipes spp (mainly A. finegoldii and A. shahii), Ruminococcus spp and Fusobacterium spp., Clostridium, lachnoclostridium | Prevotella spp (mainly P. copri), Clostridium spp, Roseburia spp, Faecalibacterium prausnitzii and Eubacterium rectale. | |
| Amanda F Barros et al 2015 | Listeria monocytogenes and Flavobacteriaceae bacterium | | Uncultured Lachnospiraceae  Bacterium and Butyrivibriocrossotus |
| Elisabetta Margiotta et al. 2020 | Citrobacter spp | | Roseburia spp |
| Kaiyu Xu et al.2017 | N/A | | N/A |
| Salguero,Maria V et al.2019 | N/A | | N/A |
| FengXia Li et al 2019 | N/A | | N/A |
| Al-Obaide, M. A. I. et al.2017 | N/A | | N/A |
| Shuanghong,Jiang et al.2016 | N/A | | Roseburia spp, and Faecalibacterium prausnitzii |
| HengzhongLun et al.2018 | N/A | | N/A |
| Wang, Siqi et al.2019 | N/A | | **ESRD:**Enterobacter, Enterococcus, Bifidobacterium, Bacteroides, Clostridium, Roseburia, Faecalibacterium and Prevotella |
| I-Wen Wu et al.2020 | Collinsella stercoris, Streptococcus anginosus, Phascolarctobacterium faecium, Lactobacillus salivarius, Escherichia coli. | | Bacteroides eggerthii, Bacteroides clarus, Bacteroides plebeius, Parabacteroides goldsteinii, Bacteroides massiliensis, Bacteroides stercoris, Ruminococcus callidus, Blautia hydrogenotrophica. |
| Xiaofang Hu et al. 2020 | N/A | | N/A |
| Zhigang Ren et al. 2020 | N/A | | N/A |
| Maria De Angelis et al 2014 | Ruminococcus obeum, Sporobacter termitidis, Subdoligranulum sp., Anaerotruncus sp., Papillibacter cinnamivorans, Clostridium bolteae, Clostridium herbivorans, Clostridium xylanolyticum, Roseburia faecis, Roseburia inulinivorans, Dorea sp., Butyrivibrio crossotus, Butyrivibrio crossotus, Eubacterium sp., Turicibacter sp., Bacteroides coprocola, Bacteroides faecis, Sutterella sp., Parasutterella excrementihominis, Escherichia coli, Escherichia sp., Proteus sp., Enterobacter sp., | | Ruminococcus gnavus, Anaerofilum sp., Oscillospira sp., Clostridium clostridioforme, Clostridium methylpentosum, Clostridium symbiosum, Coprococcus eutactus, Lachnospira pectinoschiza, Eubacterium eligens, Eubacterium oxidoreducens, Eubacterium siraeum, Lactobacillus sp., Catenibacterium sp., Eubacterium biforme, Dialister sp., Phascolarctobacterium sp., Bacteroides finegoldii, Bacteroides ovatus, Bacteroides sp., Bacteroides thetaiotaomicron, Bacteroides uniformis, Bacteroides vulgatus, Butyricimonas virosa, Prevotella copri, Prevotella sp., Alistipes putredinis, Alistipes sp., Rhodospirillum sp., Bifidobacterium sp. |
| Xiaofang Hu et al. 2020 | N/A | | N/A |
| ZhengXia Zhong et al.2020 | N/A | | N/A |
| Sibei Tao et al.2019 | N/A | | N/A |

PD, peritoneal dialysis;HD, hemodialysis. NH, nohemodialysis. BCoAT, acetate CoA-transferase.

****Table S 12. Metabolites derived from gut microbiota in advanced CKD.****

| **Study** | **Index** | **Conclusion** |
| --- | --- | --- |
| Kaiyu Xu et al.2017 | TMAO | Median plasma TMAO level in the CKD patients was significantly higher than the concentration measured in the healthy controls. |
| Al-Obaide, M. A. I. et al.2017 | TMAO | Higher level of TMAO in T2DM-CKD patients than healthy controls. |
| Wang, Siqi et al.2019 | TMAO; Acetate, Propionate, and Butyrate | TMAO in patients with ESRD was higher than that in patients with CKD 1-4 and that in healthy controls.  Acetate, propionate, and butyrate were decreased in the patients with CKD. |
| Xifan Wang et al.2020 | TMAO, pCS, Indole, Phenylacetylglutamine, and SCFA(butyric acid, propionic acid, acetic acid) | Uraemic toxin precursors and secondary bile acids were enriched in patient, while primary bile acids, SCFA and SCFA derivatives were markedly reduced. |
| I-Wen Wu et al.2020 | IS and pCS | Both IS and pCS reflected the degree of renal impairment. |

TMAO, trimethylamine-n-oxide; IS, indoxyl sulfate; pCS, p-cresyl sulfate;SCFAs, short chain fatty acids;T2DM, type 2 diabetes mellitus;CKD, chronic kidney disease.

****Table S 13. Comparison of parameters of the gut permeability.****

| **Study** | **Index** | **Conclusion** |
| --- | --- | --- |
| Feiqian Wang et al.2012. | D-lactate | Mean plasma D-lactate levels of patients with bacterial DNA in their blood were higher compared with patients without bacterial DNA and controls. |
| Stadlbauer, Vanessa et al.2017. | Zonulin | Zonulin as a marker of gut barrier integrity was not different between patient groups and controls. |
| Al-Obaide, M. A. I. et al.2017. | Zonulin | Higher level of Zonulin in T2DM-CKD patients than healthy controls |
| Terpstra, M. L. et al.2019. | D-lactate | D-lactate was elevated in the dialysis group compared to the healthy kidney donors. |

T2DM, type 2 diabetes mellitus; CKD, chronic kidney
